# Approaches to Sampling for Quality Control of Artificial Intelligence in Biomedical Research

**DOI:** 10.17691/stm2023.15.2.02

**Published:** 2023-03-29

**Authors:** S.F. Chetverikov, K.M. Arzamasov, A.E. Andreichenko, V.P. Novik, T.M. Bobrovskaya, A.V. Vladzimirsky

**Affiliations:** Head of the Sector of the Development of Systems for the Implementation of Intelligent Medical Technologies; Research and Practical Clinical Center for Diagnostics and Telemedicine Technologies of the Moscow Health care Department, 24/1 Petrovka St., Moscow, 127051, Russia;; Head of the Department of Medical Informatics, Radiomics and Radiogenomics; Research and Practical Clinical Center for Diagnostics and Telemedicine Technologies of the Moscow Health care Department, 24/1 Petrovka St., Moscow, 127051, Russia;; Leading Researcher, Department of Medical Informatics, Radiomics and Radiogenomics; Research and Practical Clinical Center for Diagnostics and Telemedicine Technologies of the Moscow Health care Department, 24/1 Petrovka St., Moscow, 127051, Russia; Head of the Group of Artificial Intelligence; K-SkAI LLC, 17 Naberezhnaya Varkausa, Petrozavodsk, the Republic of Karelia, 185031, Russia; Leading Researcher; ITMO National Research University, 49 Kronverksky Pr., Saint Petersburg, 197101, Russia;; Researcher, Sector of the Development of Systems for the Implementation of Intelligent Medical Technologies; Research and Practical Clinical Center for Diagnostics and Telemedicine Technologies of the Moscow Health care Department, 24/1 Petrovka St., Moscow, 127051, Russia;; Junior Researcher, Sector of the Development of Systems for the Implementation of Intelligent Medical Technologies; Research and Practical Clinical Center for Diagnostics and Telemedicine Technologies of the Moscow Health care Department, 24/1 Petrovka St., Moscow, 127051, Russia;; Deputy Director for Research; Research and Practical Clinical Center for Diagnostics and Telemedicine Technologies of the Moscow Health care Department, 24/1 Petrovka St., Moscow, 127051, Russia; Professor, Department of Information and Internet Technologies; First Moscow State Medical University named after I.M. Sechenov (Sechenov University), 8/2 Trubetskaya St., Moscow, 119991, Russia

**Keywords:** artificial intelligence, statistical methods, sampling, AI quality control

## Abstract

**The aim of the study** is to evaluate the efficacy of approaches to sampling during periodic quality control of the artificial intelligence (AI) results in biomedical practice.

**Materials and Methods:**

The approaches to sampling based on point statistical estimation, statistical hypothesis testing, employing ready-made statistical tables, as well as options of the approaches presented in GOST R ISO 2859-1-2007 “Statistical methods. Sampling procedures for inspection by attributes” have been analyzed. We have considered variants of sampling of different sizes for general populations from 1000 to 100,000 studies.

The analysis of the approaches to sampling was carried out as part of an experiment on the use of innovative technologies in computer vision for the analysis of medical images and their further application in the healthcare system of Moscow (Russia).

**Results:**

Ready-made tables have specific statistical input data, which does not make them a universal option for biomedical research. Point statistical estimation helps to calculate a sample based on given statistical parameters with a certain confidence interval. This approach is promising in the case when only a type I error is important for the researcher, and a type II error is not a priority. Using the approach based on statistical hypothesis testing makes it possible to take account of type I and II errors based on the given statistical parameters. The application of GOST R ISO 2859-1-2007 for sampling allows using ready-made values depending on the given statistical parameters.

When evaluating the efficacy of the studied approaches, it was found that for our purposes, the optimal number of studies during AI quality control for the analysis of medical images is 80 items. This meets the requirements of representativeness, balance of the risks to the consumer and the AI service provider, as well as optimization of labor costs of employees involved in the process of quality control of the AI results.

## Introduction

Artificial intelligence (AI) in medicine is a tool that automates routine processes such as filling out electronic records, analyzing and diagnosing medical images, doing analytics, and patient treatment planning. AI helps to reduce labor costs in the process of medical and biological activities, as well as improve the accuracy of recommendations, diagnostics, prescriptions, etc. [1-7].

When introducing AI systems into clinical practice, an important part is the quality control of AI results [8-10] in order to confirm their safety and effectiveness [11-20]. For an autonomous and adaptive AI system, the operational periodic quality control ensuring adjustment with minimal risks and a short response time is of particular value.

All types of defects in the AI operation can be conditionally divided into two groups according to the method of detection: those detected during automated and manual checks. An automated check provides quality control of the entire population over a certain period. A manual check is carried out on a limited sample and requires significant resource costs, as it involves opening and viewing images under study; clinical analysis of the original image; assessment of the AI results; management of the quality control records, etc. In this regard, the issue of sampling for quality control of the AI results is relevant and should solve at least two important tasks:

1. representativeness and correctness of the proportions of distribution (content) of the studied trait [21-24];
2. accounting for the labor costs of employees involved in the manual quality control of the AI results.

Many approaches have been proposed for calculating sample sizes in various fields of science [25-43], however, it is not possible to make a reasoned choice of one or another approach when planning a biomedical research or when introducing AI for practical use. Thus, in paper [37], it is reported that, depending on the approach used, different sample sizes are obtained.

**The aim of the study** is to evaluate the efficacy of approaches to sampling when conducting periodic quality control of the AI results in biomedical practice.

## Materials and Methods

Sampling approaches based on point statistical estimation [31, 44–47], statistical hypothesis testing — variant 1 [48-51] and variant 2 [52, 53], the application of GOST R ISO 2859-1-2007 “Statistical methods. Sampling procedures for inspection by attributes” [54-58] were considered.

The analysis of approaches to sampling was carried out as part of an experiment on the use of innovative technologies in the field of computer vision for the analysis of medical images and their further application in the healthcare system of Moscow (Russia) [59, 60]. Previously, various types of defects were found in the results of processing medical images by AI [59], which reduce the clinical and diagnostic value of the systems we studied.

In this article, a set of medical AI-processed studies over a certain period of time will be called a batch. Each batch consists of product items of the same type, class, size, and composition, processed under almost identical conditions over the same period of time. A product item is an AI-processed study (connected to the radiological information system “Unified Radiological Information Service of the City of Moscow” (URIS)) for a particular reporting period.

Each study contains the following data:

population parameters (gender and age indicators, ethnic composition, regions of residence, etc.); depersonalization data; information on medical facilities which are the source for the formation of a data set;

study characteristics (anatomical area(s); modality; projections; types of medical products — diagnostic devices; types and characteristics of research protocols);

target pathology in accordance with the International classification of diseases [61];

cases of presence/absence of pathological findings.

The quality control of the AI results is carried out repeatedly. When controlling the quality of the results of the AI systems which we study, a manual review of the studies is carried out by experts. Due to a large number (more than 1000) of studies in the batch, there is no possibility of quality control in full due to time constraints, as well as due to the small number of experts. As part of the experiment [59], it has been found that no more than 10% of defective product items are contained in the general population. This means that the entire batch for the reporting period contains no more than 10% of product items with technological defects [62]. Thus, the article describes approaches that correspond to a qualitative feature, where the proportion of cases in which the studied trait occurs is known. The volume of the general population ranges from 1000 to 100,000 product items.

The solution to the problem of the quality control of a batch with AI results was provided on the basis of selective observation, based on the concepts of general and sample population.

This article describes serial repetition-free sampling, where a selected product item was drawn from the entire volume of the general population and not returned back; thus, the probability of getting the remaining product items increased.

Calculations were performed using the PASS 15 Update — NCSS (www.ncss.com) and LibreOffice Calc (www.libreoffice.org) software.

## Results and Discussion

***The approach based on point statistical estimation*** considers the deviation of the results of a sample study from the general values. With this approach, a sample size is calculated by the formula:

n=Nt2WqNΔ2+t2Wq,

where *n* is the sample size; *N* is the size of the general population; *t* is a coefficient showing with what probability (reliability) it is possible to guarantee the reliability of the result obtained or the critical value of the Student’s criterion at the appropriate significance level (for a significance level of 0.05, the coefficient *t*=1.96); Δ is the limiting error of the indicator; *w* is the proportion of the investigated trait; *q*=(*1–w*) is the proportion where the investigated trait is absent.

Thus, when the proportion of the investigated trait (*w*) is 0.9, the level of statistical significance is 0.95, and the maximum permissible error (Δ) is 0.05, we get a sample size (*n*) equal to 138 product items.

***The statistical hypothesis testing approach (variant 1)*** involves testing the statistical hypothesis H_0_ (the quality of the batch meets the requirements) in the presence of an alternative hypothesis H_1_ (the quality of the batch does not meet the requirements). If among *n*-product items the number of defective ones (*m*) does not exceed the acceptance number *c* (*m≤c*) (the maximum permissible number of rejected sample items, which allows making a decision on the acceptance of a product batch in terms of quality), then the product batch is accepted; otherwise, it is rejected.

To select a control plan (determination of a sample), the following formula is used:

pn(m≤c)=∑m=0cpn(m),

where *m* is the number of defective items in a sample of *n*; *p_n_*(*m*) is the probability of occurrence of *m* defective product items in the *n* sample; *c* is the acceptance number.

Since in the scope of the experiment [59], the size of the entire batch exceeded the sample size by more than 10% [56], the operational characteristics were determined by the formula:

pn=Cnmqm(1−q)n−m,

where *C*is the number of combinations of *_n m_*the appearance of *m* defective product items in the sample of *n*:

Cnm=n!m!(n−m)!.

In the experimental example [59], the acceptance number equal to two product items was used, calculations were made and curves were plotted for samples of 3050, 80, 138 product items (see the Figure).

**Figure:**
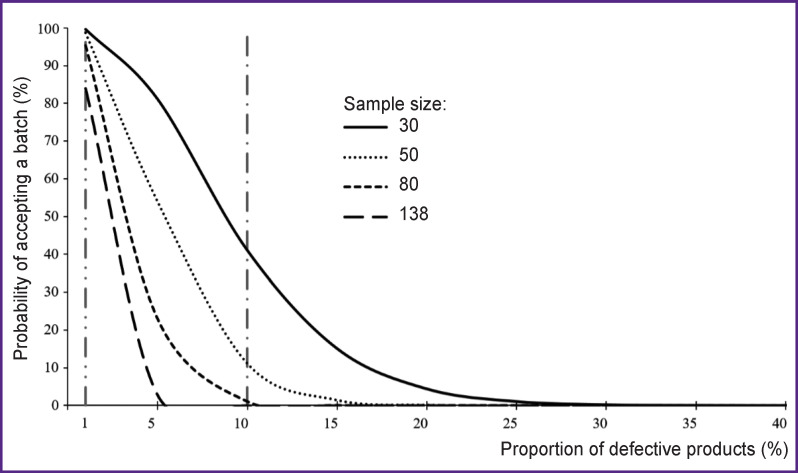
Operational characteristics for different sample sizes: vertical dash-dotted line with two dots — supplier’s risk; vertical dash-dotted line with one dot — consumer’s risk

The figure shows the consumer’s and the supplier’s risks. The supplier’s risk is the probability of rejecting a good quality batch (i.e., in the general population, the proportion of defective items of products is less than 10%). Taking into account the proportion of declared defective products from the supplier, the risk is assumed to be 1%. The consumer’s risk is the probability of accepting a low-quality batch. Accounting for the proportion of defective products identified by the consumer, the risk is assumed to be 10%.

Analyzing the data from Table 1 and taking into account the specified risks to the consumer and the supplier at the level of no more than 10% and no more than 5%, respectively, we found that the sample size, equal to 80 product items, meets the requirements of both the consumer and the supplier.

**Table 1:** Dependence of risks on the sample size

Sample size	Consumer’s risk (%)	Supplier’s risk (%)
30	41	0
50	11	1
80	1	5
138	0	16

***The approach based on statistical hypothesis testing (variant 2)*** is based on the principles of the probability of rejecting the null hypothesis; it takes into account both the supplier’s and the consumer’s risks. The null hypothesis H_0_ assumes that if the general population contains more than 10% of defective product items, then the entire batch for the reporting period contains more than 10% of product items with technological defects. Accordingly, under the alternative hypothesis H_1_, less than 10% of product items have technological defects. The probability of rejecting the null hypothesis is at least 80%.

Calculations were made (Table 2) for samples of 30, 50, 80, 120 product items with the acceptance number from zero to four (the acceptance number was limited by exceeding the risks to the consumer of over 10% or the supplier — of over 5%).

**Table 2:** Dependence of risks on the sample size and acceptance number

Sample size	Acceptance number/proportion of defects (%)	Probability of deviation from the null hypothesis (%)	Supplier’s risk (%)	Consumer’s risk (%)
30	0/0	100.0	0	8
30	1.0/3.3	36.2	63.8	37
50	0/0	100.0	0	1
50	1/2	36.4	63.6	7
80	0/0	100.0	0	<1
80	1.0/1.3	92.1	7.9	<1
80	2.0/2.5	67.7	32.3	2
120	0/0	100.0	0	<1
120	1.0/0.8	100.0	0	<1
120	2.0/1.7	98.4	1.6	<1
120	3.0/2.5	91.9	8.1	<1
120	4.0/3.3	78.8	21.2	2

Analyzing the data from Table 2 and taking into consideration the specified risks to the consumer and the supplier, as well as the proportion of declared defective items from the supplier (1%) and the proportion of defective items identified by the consumer (10%), it has been found that the sample size equal to 30, 50, 80, and 120 product items, meets the requirements of both the consumer and the supplier with the acceptance number equal to zero product items. Taking into account the proportion of defective items with acceptance numbers greater than zero, the most appropriate sample sizes were 80 or 120 items.

***The approach based on the application of GOST R ISO 2859-1-2007*** “Statistical methods. Sampling procedures for inspection by attributes” involves the determination of a sampling scheme for successive batches based on an acceptable level of quality and can be applied to various types of data or records. The acceptable level of quality is expressed as the percentage of nonconforming product items or the number of nonconformities per hundred product items.

We have considered several variants of sample size determination.

First, we addressed the “Sample size codes” table [54]. In our case, the general control level is II, special inspection levels are not used. Since our batch sizes ranged from 1000 to 100,000, the J, K, L, M codes were of interest for us. In this work, we did not plan multi-stage sampling, neither did we imply a transition to a weakened or enhanced control. In this regard, we used the data from the table “Single-stage plans under normal control (main table)” [54]: for an acceptable consumer quality level of 10% (for batches of 501 to 10,000 studies), the sample size for quality control will be equal to 125 product items with the batch acceptance number equal to zero; for batches of 10,001 to 150,000, the quality control sample size will be 500 items with the batch acceptance number equal to one.

We then addressed the table “Producer’s risk under normal control (percentage of rejected batches for single-stage plans)” [54] and obtained the supplier’s risk of 11.8% for a sample of 125 product items; 9.02% for a sample of 500 product items.

Table 3 summarizes the pros and cons of the considered approaches.

**Table 3:** Pros and cons of the approaches to sampling

Features	Approach based on point statistical estimation	Approach based on statistical hypothesis testing (variant 1)	Approach based on statistical hypothesis testing (variant 2)	Approach based on the application of GOST R ISO 2859-1-2007
Pros	Ability to determine the sample size using statistical parameters Simple math calculations Ability to choose a confidence interval sufficient for a confident judgment about general parameters based on known sample indicators	The consumer’s and supplier’s risks are taken into account Ability to calculate risks depending on the sample size, the proportion of defective product items and acceptance number Ability to visually determine the most appropriate parameters for research	The consumer’s and supplier’s risks are taken into account Ability to calculate risks depending on the sample size, the proportion of defective product items and acceptance number Statistical parameters are taken into account Ability to calculate confidence intervals for the obtained values	The consumer’s and supplier’s risks are taken into account Minimum preliminary population data are required No need for mathematical calculations No need for additional data processing software Visual determination of the most suitable parameters for research Possibility to apply different control plans at low or high batch quality levels Dependence of a sample size on high and low population sizes
Cons	Type II error is not taken into account Statistical parameters and confidence interval in biomedical research have a limited scope, beyond which the results are not statistically significant Preliminary data on general population are required	Preliminary population data are required Data preprocessing is required to determine the formulas used Fixed values of risks and proportions of defective product items are required before conducting research	Preliminary population data are required Fixed risk values are required before conducting research Cases of high item cost used for quality control are not taken into consideration The use of paid software for statistical processing is implied	Finer choice of statistical parameters is not allowed More accurate calculation of the consumer’s and supplier’s risks is not allowed Fixed values of risks and proportions of defective product items before conducting research are required
Sample size	138	80	80, 120	125, 500

The approaches to sample size calculation that we have considered have a number of advantages over the widely used approaches based on fixed or tabular values. Thus, for example, the approaches with application of ready-made tables have specific statistical input data, which does not make it possible to consider them universal [46, 63, 64].

The table based on V.I. Paniotto’s methodology [47] contains values that are calculated for specific parameters: the proportion of the trait is 0.5; allowable error — 0.05; confidence level — 0.954 (*t*=2).

The table based on N. Fox’ methodology [65] also contains a specific parameter: the proportion of the trait is 0.35.

The values from the tables [47, 65] were calculated using the formulas of the point statistical estimation described in this article above. If the input data of a biomedical study do not match the parameters of the tables, we recommend performing calculations rather than using the given tables to determine the sample size.

## Conclusion

The results of this study provide a choice of the most appropriate approaches to achieving the goals of biomedical research. The use of point statistical estimation and the approach based on statistical hypothesis testing allows for the most flexible calculation of sample sizes depending on the input parameters of the study. The use of GOST R ISO 2859-1-2007 for sampling is a priority if the experiment involves the interaction of the researcher and a third-party organization. This allows taking account of the risks and errors to both parties involved in the process.

The optimal number of studies during quality control of the AI systems that we have studied for the analysis of medical images is 80 product items. This meets the requirements of representativeness, balance of risks to the consumer and the providers of AI services, as well as optimization of labor costs of employees involved in the quality control of artificial intelligence.
